# The Role of Forensic Investigation in an Unusual Case of Patricide by a Schizophrenic Woman Involving Dismemberment of a Decomposed Body

**DOI:** 10.3390/diagnostics12071577

**Published:** 2022-06-29

**Authors:** Isabella Aquila, Matteo Antonio Sacco, Fabrizio Cordasco, Carmen Scalise, Francesco Maria Galassi, Elena Varotto, Walter Caruso, Valerio Riccardo Aquila, Pietrantonio Ricci

**Affiliations:** 1 Institute of Legal Medicine, Department of Medical and Surgical Sciences, University “Magna Graecia” of Catanzaro, Viale Europa, Loc. Germaneto, 88100 Catanzaro, Italy; matteoantoniosacco@gmail.com (M.A.S.); cordasco@unicz.it (F.C.); scalisecar@gmail.com (C.S.); ricci@unicz.it (P.R.); 2 Archaeology, College of Humanities, Arts and Social Sciences, Flinders University, Adelaide, SA 5001, Australia; francescom.galassi@flinders.edu.au (F.M.G.); elena.varotto@unict.it (E.V.); 3 FAPAB Research Center, 96012 Avola, Italy; 4 ASP, 87100 Cosenza, Italy; caruso.walter@libero.it; 5 Department of Medical and Surgical Sciences, University “Magna Graecia” of Catanzaro, Viale Europa, Loc. Germaneto, 88100 Catanzaro, Italy; valerio_aquila@hotmail.it

**Keywords:** dismemberment, patricide, decapitation, psychopathology, schizophrenia, PMCT

## Abstract

Dismemberment is characterized by the fragmentation of the body into anatomical sections. It can occur because of a murder, suicide, or accident. In the literature, there are no cases of patricide perpetrated by a daughter in which the offender performed the dismemberment. However, in this paper, we reported a case of patricide by a schizophrenic daughter that was not treated with antipsychotic therapy. Post-mortem Computed Tomography (PMCT), autopsy, and histological examinations were performed. The soft tissues were removed through maceration techniques and chemical treatment. An analysis was performed to study the bone margins and clarify the weapon and manner of death. This investigation, which used radiological and histological studies, helped to assess the vitality of the injuries. The purpose of the study is to discover the weapon used, the cause, and the manner of death, with particular interest in this case due to the dismemberment. Moreover, we emphasize the correlation between patricide, dismemberment, and a lack of antipsychotic treatment in patients with schizophrenia.

## 1. Introduction

Dismemberment is a method whereby the murderer, after killing the victim, uses a weapon to cut the body into small pieces. The term dismemberment is also related to human mutilation, which is defined as “the act of depriving an individual of a limb, member, or other important part of the body; or deprival of an organ; or severe disfigurement” [1]. The act of dismembering a body could take different meanings and may have different purposes. The reasons for the implementation of “dismemberment” can be: the need to conceal the corpse and, also, make impossible the ability to identify the victim (defensive mutilation) [2] or, especially in cases of sexual murder, it can be the reason for the crime (offensive mutilation) [3,4] so, in these events, the dismemberment is the result of impulsive and aggressive actions against the corpse of the victim wherein removing body parts becomes a demonstrative act. Also, dismemberment could be a form of cannibalism (at least Neanderthals dismembered their dead relatives to use the body parts not only as food but also as special tools). Moreover, the literature reports another two categories: aggressive mutilation, which represents a way to kill and severing the body parts is the cause of death; or necromaniac mutilation, which is occurs, for example, when the offender collects parts of a buried corpse for their own pleasure [5].

In cases of dismemberment, there is usually a familial or interpersonal relationship between the murderer and the victim. Many studies have shown a link between the reason for the crime and the implementation of dismemberment by the perpetrators [6,7,8,9]. In particular, Puschel and Koops have carried out a classification according to the motives of perpetrators, consisting of three groups: sexual perversion, psychosis, and other psychiatric disorders. The first group mutilates the body mainly by cutting the genitals or breasts and, in these cases, usually the cause of death was due to strangulation [10]. The second and third group include aggressive and necromaniac mutilation. In these two groups, the fundamental role of the forensic pathologist is to distinguish the vital injuries from the post-mortem lesions. Dismemberment can occur in life or after death. In some cases, hemorrhage due to the dissection of the corpse is the cause of death, while in other cases, the dismemberment occurs after death due to some other cause. In many forensic cases, it is complicated to discern the cause of death. In fact, it is sometimes an unresolved question for forensic pathologists and investigators.

Below, a very peculiar case of patricide with dismemberment of the body and decapitation by his daughter is reported. The findings from the autopsy and histological and radiological investigations gave useful information to define the cause of death, the vitality of the sharp injuries on the different tissues, and the manner of death. The purpose of this work is to evaluate, through the presentation of a case of patricide and a review of the literature, the contribution of forensic science in solving cases in which the body is dismembered in order to answer fundamental questions, such as what weapon was used, what was the cause, and what was the manner of death? In particular, we stress the importance of the analysis of macroscopic bone margins with radiological study and histopathological examination regarding the injury’s vitality.

Moreover, we emphasize the role of psychiatric illness and, especially, the influence non-pharmacological treatment has on the aggressor in the genesis of patricide.

## 2. Case History

**Figure 1:**
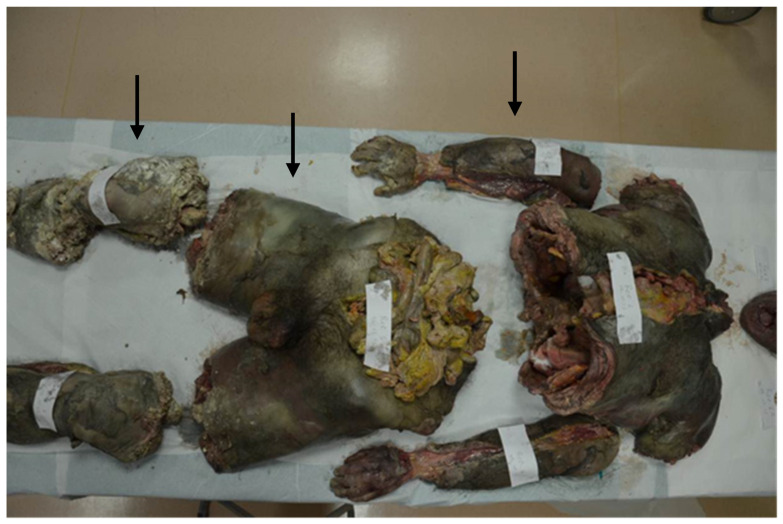
The body segments were analyzed and subjected to an anthropological investigation. There were no deposits of eggs found nor any presence of larvae, which assumes there was a prolonged exposure of the body before concealment.

The victim was a seventy-two-year-old man. He was a retired teacher and a widower, and he lived in his apartment with his thirty-eight-year-old daughter. The woman suffered from psychiatric disorders, she received a schizophrenia diagnosis when she was young and was not treated with drugs. The witnesses claimed that the relationship between the father and his daughter was conflictual and characterized by constant bickering. The old man disappeared for about a month, so his apartment was subjected to a judicial inspection with detailed video-camera recording. During the investigations, the police found seven boxes in the entrance of the apartment. In these boxes they found many body segments, still covered by clothing with laces and ribbons, surrounded by lime, and covered with cellophane. In the environment there was no blood or larvae (Figure 1).

Box n° 1 and n° 3 contained bloodstained tools used to dismember the body; box n° 2 contained the anatomical portion of the thorax and abdomen. Box n° 4 contained the right foot and the upper limbs. Box n° 5 contained the decapitated head. Box n° 6 contained portions of the lower limbs. Box n° 7 contained the anatomical portion of the abdomen–pelvis with the proximal portion of the lower limbs. Furthermore, examination of the crime scene pointed out a hatchet and an ax covered with a sheet. Investigations with Luminol showed the presence of human blood at the cutting and blunt edge of the ax. When the victim’s daughter was interviewed, she confessed only the dismemberment, claiming to have found the body of her father already dead and divided into two parts.

The problem for investigators was to determine the vitality of the injuries inflicted on the body of the elderly man, the time of death, and the manner in which the murder occurred.

## 3. Materials and Methods

The body segments were analyzed through an anthropological survey and then a MSCT total-body 3D with a 64-slice MSCT system (Aquilion CX 64, Toshiba Medical System, Tokyo, Japan) was carried out in order to obtain a 3D reconstruction of the bone fragments to carry out an accurate macroscopic analysis. At the conclusion of the PMCT, an autopsy was performed and tissue samples were taken in order to perform a histopathological investigation of the organs. The tissue samples were preserved in formaldehyde that was diluted to 10%. For all the samples, the methods and systems of classical histology by paraffin embedding and hematoxylin-eosin staining were used; descaling treatment was also adopted for the fragments of the parietal bone tissue by immersion in long latency in a mixture of hydrochloric acid, nitric acid, and sulfuric acid in order to obtain adequate descaling, which allowed the passage of bone tissue to the rotary microtome. Successively, the head was placed in a cold room at 2 °C for further anthropological examination and skeletal reduction. Following this, the soft tissues of the head were skinned with the use of a scalpel. The soft tissue dissected was analyzed on the periosteal side. The skeletal reduction was achieved by maceration techniques [11] for two days at a temperature of 55–60 °C. The human remains were subsequently so treated:

- with water and sodium hypochlorite 5.5% for 15–30 min [12];
- final washing with water and alcohol to stop the chemical process and prevent corrosion of the bone.

The remains so treated were cleaned with a foam pad and then they were photographed. Finally, genetic investigations were performed on a sample of each box and a comparison was performed with a biological sample belonging to the woman’s father.

## 4. Results

### 4.1. PMCT

**Figure 2:**
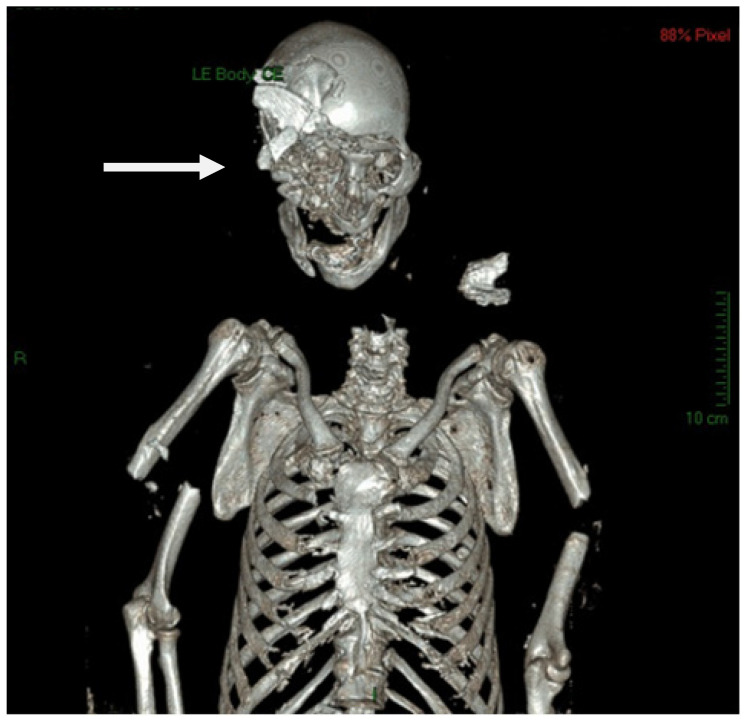
The PMCT showed multiple fractures on the head facing inwards and net margins inflicted by an object with a cutting edge.

**Figure 3:**
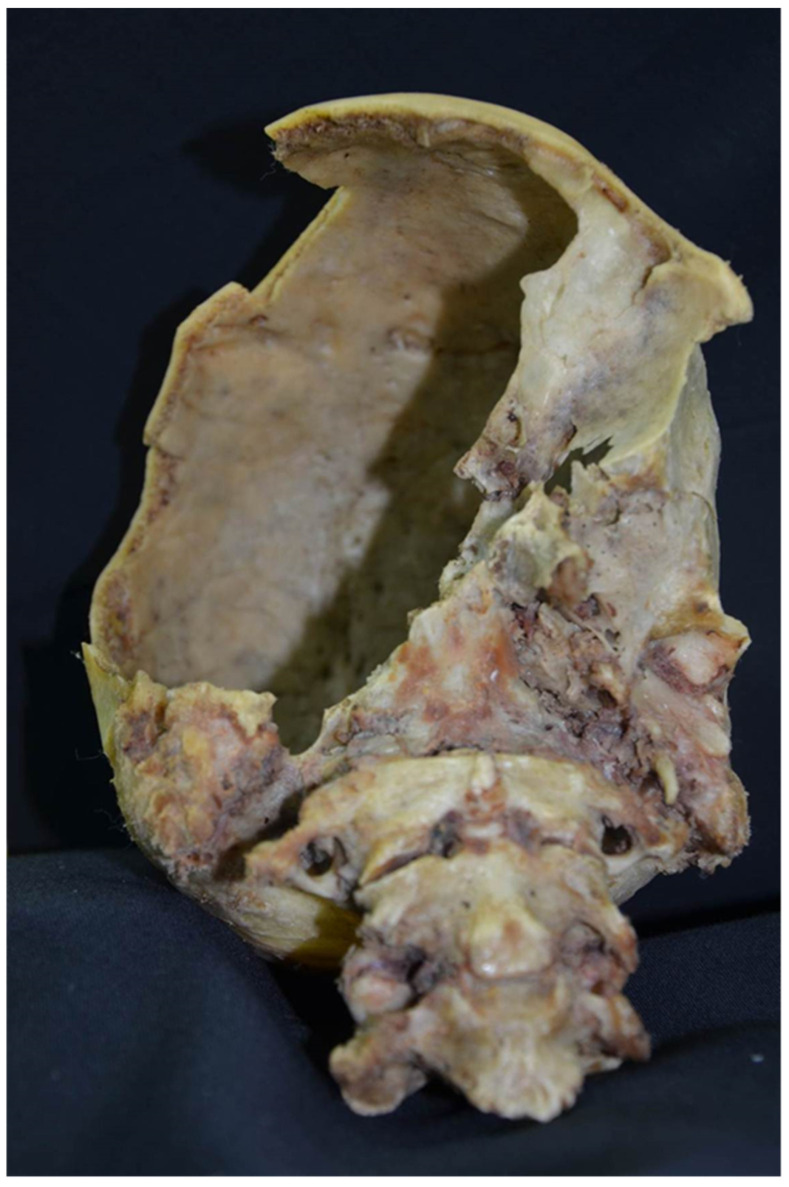
The skeletal reduction findings showed that the skull had fractures on the frontal, temporal, parietal, and occipital right side. The margins of all the fractures of the head showed macroscopic characteristics of vitality.

**Figure 4:**
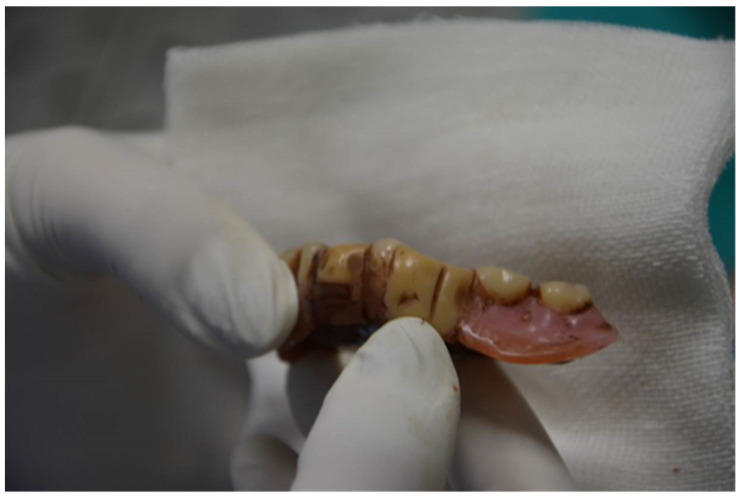
Two dental prosthetic elements of the mandibular arch were scratched on the front surface with non-serrated margins, such as a negative impression of the sharp blade of the medium used.

The PMCT showed multiple fractures on the head facing inwards and net margins inflicted by an object with a cutting edge (Figure 2, Figure 3 and Figure 4). Instead, other areas of the skull showed jagged bony edges, which correspond with the use of a blunt object. The analysis of other sectioned anatomical districts showed many small fragments that were compatible with the use of an object with a sharp edge with a high weight.

### 4.2. Autopsy Findings

At autopsy, the body was identified, and the external examination revealed the presence of nine dissected body segments. The skull on the right side was broken and there was leakage of the brain, which was completely liquefied. The left side of the face had ecchymosis, which was particularly highlighted in the left frontal and zygomatic area. The hands had areas of skin maceration. The survey noted a translocation of the abdominal organs from their natural site. An examination of the bone margins of the anatomical sections did not show the presence of vital features. The complete absence of infiltration and bleeding permitted us to establish that they were post-mortal injuries.

### 4.3. Skeletal Reduction Findings

It was found that two dental prosthetic elements of the mandibular arch were scratched on the front surface with non-serrated margins (such as a negative impression of the sharp blade of the medium used). Instead, the maxillary arch prosthetic presented no scratching. The bone elements of the hard palate were fractured. The skull presented fractures in the frontal, temporal, parietal, and occipital right side. The margins of all fractures of the head showed macroscopic characteristics of vitality. On the bones, it was noted that there was a rupture of the right styloid process and a fracture into three points of the mandible on the right branch. The third cervical vertebra appeared tangentially scratched on the lower surface. It also noted that there was a fracture of the hyoid bone on the right side, 5 shards with edges and linear vitality belonging to a crown, and 14 fragments belonging to the splanchnocranium.

### 4.4. Histopathological Investigations

Putrefaction was distributed to all the internal organs; the lungs were putrefied and the heart seemed to be in advanced degenerative degradation. The margins of the head bone highlighted the presence of edema and intra-osseous hematic effusion with vital features, which was in contrast to other parts of the body.

### 4.5. Genetic Investigations

The investigations proved that all biological samples found in the boxes belonged to a unique victim, which corresponded to the woman’s father.

## 5. Discussion

### 5.1. Patricides

The term patricide, specifically, means the homicide of the father figure. It is estimated that it represents 1–4% of all homicides and 20–30% of homicides committed by psychotically ill individuals. The rarity of the phenomenon creates significant difficulties for data collection [13,14,15,16]. A study with a larger number of cases was carried out by Devaux et al. who reviewed 61 cases of patricide committed between 1958 and 1967 [12]. Forensic psychiatry evidence has demonstrated that patricide is predominantly a male-on-male (son-on father) crime, though this predominance was reduced in recent decades [14,17]. The mean age of the victims varies from 50 to 60 years, while the mean age of the perpetrator varies from 20 to 30 years [18,19]. Bourget et al. have shown that patricide by daughters is extremely rare [20]. Generally, the most used weapon for the crime is a knife, even if the choice is related to its availability, e.g., in the USA, murderers tend to use firearms because they are more common [18,19,20,21].

Patricides are divided into two categories: adolescent patricides, which are often linked to stories of abuse or mistreatment, and adult patricide, which is usually perpetrated by individuals affected by psychiatric disorders with conflictual relationships [14,15,16,17,18,19]. The reported case belongs to the second category; in fact, it occurred during adulthood due to personal conflicts with the subject with mental disorders. Some studies have analyzed the reasons for patricide and have revealed that patricide could increase in the presence of mental disorders or in the absence of adequate treatment in subjects with psychiatric disorders. So, the homicidal behavior may represent an expression of the mental illness [18,19,20]. Psychotic disorders predominate and account for 58–100% of reported cases among offenders with mental illness. Paranoid schizophrenia appears to be the most prevalent pathology, representing approximately 40–80% of psychotic patricides [14]. Moreover, the attackers are usually suffering from schizophrenia with symptoms of active psychosis at the time of the crime and persecutory motivation is often evident [18,19,22,23,24,25,26,27]. Even in this case report, the daughter suffered from schizophrenia and was not treated with drugs. This confirms the correlation, which has been highlighted in the literature, between mental disorders—above all schizophrenia—lack of therapy, and patricide [18]. For this reason, it is important to identify this predictive factor in order to intervene in the social, family, and therapeutic setting in hopes of avoiding these events—although they are rare [13]. Alcohol and drug abuse, financial dependence by the family, and the lack of interests and activities outside the family are precipitating factors. The involved individuals experience the sensation of feeling trapped, and this contributes to the creation of family conflicts. Moreover, the review of the literature shows that patricide is usually committed in the victim‘s house [18,19,24,25,26,27,28,29,30]. It is probable that the choice of location of patricide occurs for two factors: the attacker has more time and greater security in order to conceal the corpse and the traces of murder. Also, in the case reported, the murder happened in the house where the father and daughter lived.

Hellen et al. carried out a psychosocial analysis of ten cases of homicides committed by women with an urban mid-European background [31]. All the reported cases had three common features: perpetrators were socially isolated and without support, they killed their victims at home, and they did not plan the killings. A history of burden of caring for family members, previous abuse of the perpetrator by the victim, pathological personality traits of the perpetrator, as well as helplessness, dependence, and first-degree kinship of the victim with the perpetrator were other characteristic features detected in the cases investigated. According to the literature, the authors found that women who kill show more psychosocial stressors throughout life than their male counterparts (e.g., negative family environment, previous physical and/or sexual abuse or history of family violence, extreme life conditions at the time of the crime). Furthermore, they are often unemployed, and they less frequently have previous criminal activity. In the literature, women were more likely to suffer from a mental disorder at the time of the crime than men. This finding is very important considering that woman tend to kill under stressful situations and those showing compulsive behavior have a higher risk to overtax themselves than women without these personality traits. Offenders in this study were regularly facing challenging social situations and these four affected women showed a marked lack of social integration. With the exception of the psychotic perpetrator, the authors observed two main motives for the murders: the first was the burden associated with the care of the dependent victim (be it a child or an ill family member); the second motive was a response to past victimization or self-defense for acts of violence. The authors conclude that in their study, all women were socially isolated, did not seek help—except for one woman—and did not plan their deeds. To prevent this tragic event, knowledge of the risk factors is important, but prevention efforts are hard to propose precisely because of the social isolation and lack of premeditation [31]. The review of the literature showed that the subjects committing these crimes are unemployed or have a part-time job and are not married usually [13]. Generally, the most used weapon is a knife, even if the choice is related to its availability within various communities, e.g., in the USA, murderers tend to use firearms more often than in other countries where sharp instruments are more common [18,19,20]. Many authors claim that perpetrators consider the gender, and so the strength of the victim, in the choice of the weapon that is used. For this reason, they tend to use firearms to kill males and sharp weapons for females. However, it is very important to consider the context in which the crime is committed. Indeed, most cases of patricide are not premeditated and the instruments used to kill are the most accessible [19].

### 5.2. Dismemberment

The review of the literature showed a few cases of dismemberment. These cases concerned homicidal, suicidal, and accidental manners of death. Regarding dismemberment in homicide, Hakkanen-Nyholm et al. examined the sociodemographic characteristics of the offenders, their crime history, and the presence of psychopathology and or psychopathy; they concluded that educational and mental health problems in childhood, in-patient mental health contacts, self-destructiveness, and schizophrenia were significantly more frequent in offenders that were guilty of mutilation [30]. The high prevalence of developmental difficulties and mental disorder in this kind of offender needs to be recognized [30]. Konopka et al. described that homicides ending with corpse dismemberment are most commonly committed by a person close to the victim. They are generally performed at the site of homicide and in the place inhabited by the victim, the perpetrator, or shared by both [32]. In our case, the dismemberment occurred after the murder and, in agreement with the studies mentioned, it was committed by the victim’s schizophrenic daughter in the house in which they lived. Morcillo Mendez et al. have demonstrated this method (dismemberment) on human remains found in Colombian armed conflicts [33]. In this study, the dismemberment was the cause of death due to hemorrhage and it was also used as a manner of torture. The authors showed, through the autopsy findings, the characteristics of the weapon used (cut marks), and signs of ligature used around the ankles and wrists. Among books, Black et al. proposed an analysis on the act of dismemberment in criminal contexts by offering a psychological investigation of the perpetrator and describing the main issues with forensic investigation in these difficult cases [34]. Also, Ross et al. deepened this topic, describing the complex trend of the phenomenon in different countries of the world with a multidisciplinary approach, also offering an anthropological and forensic discussion about the features of the injuries associated with dismemberment [35]. Regarding dismemberment in suicide, it has been observed that dismemberment and, in particular, decapitation is also a consequence of suicidal acts. Tsokos M. et al. described ten cases, of which eight included being run over by a train and two included cases of hangings [36]. In these cases, the macroscopic observation showed the vitality of the lesions. In particular, the decapitations/dismemberment from railway-related suicide are often the subject of discussion when considering the manner of death [37]. For example, in their study, Kendell et al. clarify the manner of death in an unusual case of suicide by Light Rail Transit in which lesions on the deceased’s remains, at a macroscopic level, were similar to those that would be caused by sharp force trauma [38]. Hanging could result in a complete decapitation as demonstrated in the case described by Dedouit et al. The authors showed a case of complete post-hanging decapitation, which underlines the importance of differential diagnosis with post-homicidal decapitation [39]. In addition, there are studies in which very rare suicidal procedures due to cuts and multiple stabbings are described. In this group, Obenson K. et al. observed a case in which lethal sharp force injury was inflicted by a homemade guillotine [40]. Ventura et al. described one suicide case that occurred by multiple sharp weapon wounds in which it was fundamental to the forensic investigation to differentiate a suicidal act from a homicidal one [41].

Decapitation can also be practiced with the aim of keeping a trophy or fetish of the victim. It also may be a dehumanizing act perpetrated in cases of offensive dismemberment when there is a close relationship between the murderer and the victim. In the literature, four categories are indicated. The first category is “defensive mutilation”, where the motive is to dispose of the corpse or make it more difficult to identify. The second category is “aggressive mutilation” in which mutilation of the body may involve the face and the genital organs. The third category is “offensive mutilation”, wherein it is possible to detect two principal motives: a necrophiliac urge to kill and to carry out sexual activities with a dead body, and the mutilation can be prior or subsequent, or a sexually sadistic need to carry out sexual activities while inflicting pain, injuries, or killing so the mutilation can be initiated when the victim is still alive and continued after death. The last category is “necromaniac mutilation”, which is carried out on a dead body after killing; in this case, some parts of the body may be used as a trophy or fetish [42,43]. There is another classification that is distinguished as “expressive” mutilation, which is when the victim represents a symbolic target, towards which the communication or satisfaction of the psychological needs of the perpetrator are directed. As well, there are “instrumental” mutilations, where the main aim of the perpetrator is to achieve some advantage [43] For what concerns the relationship between dismemberment and psychiatric disease, there are few studies available. In a study, a link was described between aggressive and defensive mutilations and alcohol/drug addiction, including previous psychiatric and criminal records, whereas in the case of sexual crimes, previous criminal records are frequently present along with anxiety, schizophrenia, or drug addiction [5]. Furthermore, although criminal mutilation is considered sadistic and cruel, it has rarely been studied with regard to psychopathic personality disorder. Psychopathy is defined as a constellation of affective, interpersonal, and behavioural characteristics including impulsivity; irresponsibility; shallow emotions; lack of empathy, guilt, or remorse; pathological lying; and the persistent violation of social norms and expectations. Decapitation after death may be associated with a number of quite different scenarios and animal predation is one of the more commonly occurring events. Post-mortem mutilation may also be a strong indicator of homicide if there is no obvious explanation from a scene as to how injuries occurred. The reasons for post-mortem decapitation are varied and may be an attempt to reduce the body into more manageable fragments and to render it less identifiable. Alternatively, cutting a body after death may be a manifestation of significant psychiatric illness in the perpetrator that may be associated with ritualistic and sexual activities.

Finally, in the literature, cases of suicidal dismemberment perpetrated using a saw have been described; Grellner et al. showed two cases of women who died due to deep cut injuries on the neck [44]. Furthermore, Schyma et al. reported a case of murder–suicide with a chain saw. In accidental injuries from a chain saw, the lesions occurred on the left side of the body. However, in this case, the characteristics of the cut injuries have demonstrated the dynamic of a suicide occurred by using the chain saw on the neck [45].

Dismemberment has also been described as a result of accidents, particularly motorcycle accidents. Zoja et al. described a case report regarding a young man that lost control of his motorcycle and was thrown about 20 m, hitting his head against the barrier separating a tramline from the road. The resulting trauma caused his decapitation, the only fatal wound ascertained by various forensic investigations [46].

Therefore, in these cases, decapitation and dismemberment are more complicated to distinguish from murder.

### 5.3. Analysis of the Injuries

In all studies, the importance of wound analysis was highlighted; in particular, the characteristics of vitality and compatibility with the medium used for the dismemberment were detected. The most widely used medium is a sharp weapon. Thompson et al. have highlighted the consequences of sharp-force trauma on the human body by analyzing the stab marks on bones. In this study, they pointed out the importance of the interpretation of sharp-force trauma in order to provide information on the position of the victim to the offender, the handedness of the aggressor, and the manner of death.

Hard tissue (such as bone) represents the part of the body that best retains the imprint of the cutting edge used by the attacker. This happens even when the decomposition and dissolution of the other tissues occur, and it is especially useful for distinguishing stab marks made by a serrated and non-serrated blade through the production of a “Y”-shaped or “T”-shaped incision, respectively [47,48]. In the literature, there are several studies that highlighted, in these cases, the importance of anthropological analysis [38] combined with new methods. Porta et al. presented six different cases of dismemberment that occurred in Milano, Italy, between 1999 and 2011, in which, through the macroscopic-morphological analysis of bone lesions (which remains a crucial step in forensic investigations) and microscopic methods, e.g., SEM-EDX (Scanning Electron Microscopy combined with Energy Dispersive X-ray analysis) and stereomicroscopy, it was possible to establish the manner of death and dismemberment and the identification of the tools used in order to distinguish dismemberment and disarticulation and to make a hypothesis about the criminal context [5]. Capuani et al., in their study on hacksaw marks on bone, used epifluorescence macroscopy, a technique that, without destruction of the sample, allows the researcher to establish the features of the bone imprints in order to identify the unique blade responsible for the lesions [49]. As well Muccino et al. highlighted the decisive role of SEM-EDX in the identification of the weapon used in a case of patricide [50].

In our case, the data analysis and comparison of the results made it possible to claim that the offender was in a position of dominance with respect to the victim; the victim certainly had their wrists and ankles tied in a state of physical inferiority. From the analysis of the weapon used, it was possible to affirm that the attacker did not need extreme physical force to commit the crime because the sharp weapon had mutilating properties. The analysis of bony evidence has shown the presence of “T”-shaped incisions from a non-serrated cutting edge. It was possible to show this data through maceration techniques and skeletal reduction. The analysis of the bony margins is important in both decomposed and fresh corpses. In our case, the corpse was putrefied because about 30–40 days had elapsed since the death.

The bone tissue responds to external stress (tension, compression, torsion, bending, and shearing forces) through reversible and irreversible changes. Therefore, the study of bone fractures and their edges allows us to analyze the impact force and medium used [51,52,53].

In this case report, the examination of head bone injuries allowed us to claim that the trauma occurred when the subject was still alive. The histopathological examination of the cranial bone showed the presence of edema with intra-osseous hematic effusion, while the investigation of the other dismembered body parts didn’t show this histopathological data.

The characteristics of all the injuries detected on the bone remains were consistent with the use of a single weapon with sharp, non-serrated edges, an imposing mass, and contemporary blunt action. The analysis of the weapon found on the crime scene allowed us to detect a cutting edge and an opposite blunt surface and then to hypothesize its correspondence with the injuries.

Finally, the analysis of the bone margins permitted us to detect the manner in which the victim was hit. The sharp and blunt weapon has been used both with tangential and perpendicular force due to the presence of cuts with longitudinal and transverse direction. For this purpose, the radiological procedure (MSCT 3D) enables the analysis of bone edges and their reconstruction, even in cases of dismemberment [54,55].

The MSCT has allowed us to reconstruct the bone introflection margins and their morphology. In particular, the radiological investigation has detected the presence of multiple fragments at the upper and lower limbs due to the increased force in the trauma inflicted.

It often happens that, in addition to the dismemberment, there is the decapitation of a corpse. This finding makes it even more difficult to determine the cause and manner of death.

Dogan et al. [56] described in their case the place where the matricide was reported. Even in our case, the victim was the parent (father) of the offender and the dismemberment was performed in her house, which was also the site of the homicide. As in the case of Dogan et al., even in our study the weapon used for dismemberment and decapitation was an edged weapon with blunt action (slash); although the dismemberment involved all anatomical portions, and it was extremely symmetrical (right side than the left side). Also, in this case of dismemberment, there was the decapitation of the body.

After all these observations, in accordance with the literature, we can conclude that in order to establish the manner of death, it is essential to perform a careful diagnostic approach through:

- the analysis of the setting and the location where the crime occurred;
- the collection of psychiatric data of the probable offender (including the presence of antipsychotic drug treatment) in order to intervene in the social and family setting (psychological autopsy);
- the investigation of sociodemographic data about the victim and aggressor;
- the collection of evidence at the crime scene and, in particular, research of the weapon;
- the macroscopic analysis of the bone margins with radiological and histopathological studies regarding the vitality of the injuries;
- the evaluation of the compatibility of the sharp weapon found with injuries;
- genetic investigations on the body fragments, especially when recognition is difficult due to putrefaction.

## 6. Conclusions

In conclusion, in this case, the forensic investigations described led to the case being solved. Bone evidence allowed us to determine the cause and manner of death. In fact, the victim died due to traumatic brain injury inflicted by a blunt body consisting of the blunt part of the ax, which was found at the scene. The dismemberment occurred after death by using the cutting edge of the same weapon. The manner of death was typically homicidal. This conclusion resulted from the analysis of the crime scene and, especially, from the presence of multiple vital injuries on the head with post-mortal lesions on other parts of the body (due to dismemberment). Further, the case showed a unique relationship between patricide, dismemberment, and a lack of antipsychotic treatment in the murderer (daughter) with schizophrenia. In this case, the forensic evidence helped to offset the innocence declared by the daughter and her reconstruction of the events that occurred.

## Data Availability

Not applicable to this article as no datasets were generated.
